# Four-year analysis of cataract surgery rates in Shanghai, China: a retrospective cross-sectional study

**DOI:** 10.1186/1471-2415-14-3

**Published:** 2014-01-10

**Authors:** Mingming Zhu, Jianfeng Zhu, Lina Lu, Xiangui He, Rong Zhao, Haidong Zou

**Affiliations:** 1 Shanghai Eye Disease Prevention & Treatment Center, Shanghai, China; 2 Department of Ophthalmology, Shanghai First People’s Hospital, Shanghai Jiaotong University, Shanghai, China

**Keywords:** Cataract surgery rate, Districts, Medical institutions, Restricting factors, Shanghai

## Abstract

**Background:**

The cataract surgery rate (CSR) is a critical index used to show that cataract blindness is being eliminated. It is considered to be tightly connected to social economic development; however, it is still extremely low in developing countries such as China. Although Shanghai is the most economically developed city in China, its CSR and the obstacles for increasing its CSR have not been previously evaluated.

**Methods:**

A retrospective cross-sectional study was conducted. By analyzing the data in the “Shanghai Cataract Operations Database” from 2006 to 2009, the CSR in Shanghai was calculated. The numbers of cataract surgeries between urban and suburban districts as well as among various medical institutions were compared.

**Results:**

The CSR in Shanghai increased from 1741 in 2006 to 2210 in 2009, reflecting a 26.94% improvement. Phacoemulsification was the most frequent surgical choice for cataract removal, accounting for 94.93% of total cataract surgeries by 2009. In addition, by 2009, the CSR in urban districts had reached 5468, but only 532 in the suburbs. During 2009, cataract surgery records in 68 district hospitals, 23 medical centers, and 6 private hospitals comprised 32.05%, 52.33%, and 15.62%, respectively, of the total. There was a nearly 3.3-fold increase in the number of surgeries performed in private hospitals in the past four years. Furthermore, the average number of cataract surgeries per doctor that took place in private hospitals per year reached 207, which exceeded the average of 145 that took place in medical centers.

**Conclusions:**

Until 2009, the CSR in Shanghai remained below the rates of social development and fell short of targets suggested by the World Health Organization (WHO). Furthermore, increasing the CSR in the suburbs as well as in district hospitals is an important issue that needs to be addressed.

## Background

The World Health Organization (WHO) estimates that about 285 million people worldwide are afflicted with visual impairment, 39 million are blind, and 246 million have low vision [1]. It is estimated that as much as 80% of blindness is avoidable [1], defined as being preventable or treatable with interventions. Among all factors leading to blindness, cataracts, which are completely curable by surgery but not preventable, are the leading cause of blindness and account for 51% of total blindness cases [1]. The cataract surgery rate (CSR), which is defined as the number of cataract surgeries per million people per year, is a critical index used to show that cataract blindness is being eliminated; the rate is higher in well-developed countries, while it is still very low in some parts of Africa and Asia [2].

Generally speaking, the CSR is closely related to an individual’s financial ability to afford cataract surgery [3]. Shanghai enjoys a booming economy with a gross domestic product per capita (GDPPC) of US$9,318.50 in 2006, which was more than 10-fold that of India [4]. However, by 2006, the CSR in Shanghai was just 1741 (see details below), only about half that in India (4067) [2]. The aim of this study was to analyze the implementation of cataract blindness prevention and determine factors that reduce the CSR in this city, thereby providing insight into measures that may help eliminate avoidable cataract blindness in other countries and regions.

## Methods

**Figure 1:**
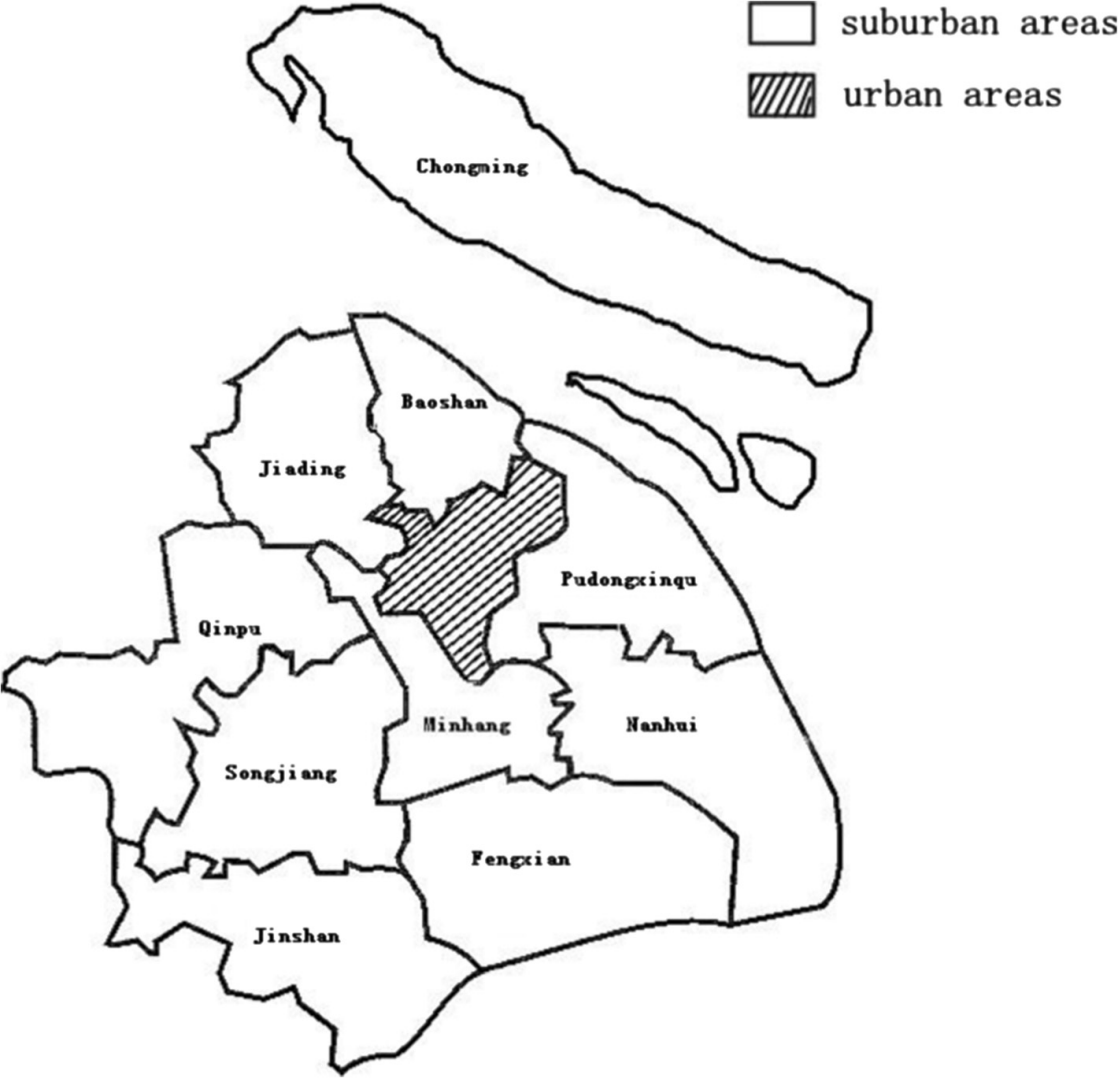
Distribution of the urban and suburban areas in Shanghai.

Shanghai, a city of 19.21 million people with a GDPPC of US$11,560 in 2009, is located in the eastern part of China. It is comprised of 19 districts, which include the Huangpu, Luwan, Xuhui, Jingan, Zhabei, Putuo, Yangpu, Changning, Hongkou, Minhang, Baoshan, Jiading, Pudongxinqu, Songjiang, Fengxian, Qingpu, Jinshang, Chongming, and Nanhui districts. According to city planning criteria, nine districts, Huangpu, Luwan, XuHui, Jingan, Zhabei, Putuo, Yangpu, Changning, and Hongkou, are defined as urban areas, while the others are in the suburbs (Figure 1).

Beginning in 2003, with the implementation of government policies, data for all cataract patients who had operations in Shanghai were recorded on a “Cataract Operations Registration Form,” which included the following items: name, sex, age, home address, cataract classification, detailed ophthalmological exam information (such as naked eyesight, optometry, tonometry, etc.), cataract surgeon, surgical management, intraocular lens (IOL) implantation or not, operation cost origin, operation outcome (mainly 1st–3rd postoperative day corrected visual acuity (VA)), etc. All cataract surgery units were requested to complete the registration form before the 5^th^ day of the next month and report the data to the Eye Disease Prevention Center in the corresponding region. Then, the Eye Disease Prevention Centers in 19 districts gathered the data from registration forms into “A Database of Cataract Surgery in Shanghai” and reported the data to the Shanghai Eye Disease Prevention & Treatment Center twice a year. The information obtained during the first half of the year (Nov. 1st of the previous year to Apr. 31st of the current year) must be reported by May10th, and that obtained in the second half of the year (May 1st to Oct. 31st of the current year) must be reported by Nov. 10th.

After data for one year had been collected, a random inspection in 4 regions of the 19 total districts was performed by the Shanghai Eye Disease Prevention & Treatment Center to review the quality of data. During the inspection, 10% of case records were randomly selected from all patients undergoing operations in a given district to assess the quality of data, including the missing report rate and the fault report rate in the reported data. The results showed a highest missing report rate of 3.50% and fault report rate of 5.74% in the data reported in Xuhui district (2006) and Changning district (2007). These results showed that the data collected was highly reliable.

However, since 2010, all data for cataract surgeries must be reported to the “Ministry of Health of the People’s Republic of China” directly. Currently, because the accuracy and integrity of data in Shanghai cannot be examined, we analyzed only the data from 2006 to 2009.

A few explanatory notes regarding this study are in order: 1) The main outcome measure was the CSR, which was calculated as follows: the CSR equals the number of cataract surgeries in Shanghai (or a district of Shanghai) in a given year divided by its resident population (or population of the district) in that year (in millions). Home addresses are useful for us to identify where our operation patients live, i.e., urban or suburban, when we calculate the CSR in these two areas. For instance, if a patient who lived in Hongkou district had his cataract surgery in Changning district, his operation contributed to the number of cataract surgeries in Hongkou district when we calculated the CSR. 2) The surgical techniques mentioned in this study include phacoemulsification (Phaco), extracapsular cataract extraction (ECCE), and others, such as intracapsular cataract extraction. 3) The IOL implantation rate (IOL%) was defined as the proportion of all cataract operations with intraocular lenses. 4) The 1st–3rd postoperative day corrected Snellen VA was the main evaluation index for the outcome of surgery. In a few areas of Shanghai, patients are kept in hospital for one or two days after surgery; while in most hospitals, patients are discharged the day after surgery. 5) In shanghai, a surgeon was not allowed to operate in more than one hospital before Dec 15, 2011. 6) All data for the population, GDP, and per capita disposable income in this study were obtained from the Shanghai Statistical Yearbook from 2007 to 2010.

## Results

A. Cataract surgery survey in Shanghai from 2006 to 2009 (Table 1).

**Table 1:** Analysis of cataract surgeries in Shanghai, 2006 to 2009

	**No. CS**	**Population**	**CSR**	**Surgical methods (%)**			**IOL%**	**VA outcome**	
		**(million)**		**Phaco**	**ECCE**	**others**		**A%**	**B%**
2006	31604	18.15	1741	89.02	9.92	1.06	98.64	98.58	87.45
2007	34563	18.58	1860	90.92	8.06	1.02	98.54	98.18	86.03
2008	38748	18.88	2052	93.78	5.40	0.82	98.78	97.38	88.05
2009	42459	19.21	2210	94.93	4.07	1.00	99.06	97.90	87.48

*Abbreviations* used: *No. CS* the number of cataract surgeries, *Phaco* phacoemulsification surgeries, *ECCE* extracapsular cataract extraction surgeries, *A%* percentage of patients with 1st–3rd postoperative day corrected Snellen visual acuity (VA) ≥ 0.05, *B%* percentage of patients with 1st–3rd postoperative day corrected Snellen VA ≥ 0.3.

The CSR in Shanghai increased steadily from 1741 in 2006 to 2210 in 2009, an increase of 26.94%. Phacoemulsification was the primary choice for cataract removal, with the rate rising every year. From 2006 to 2009, the IOL implantation rate was greater than 98%, and more than 86% of patients had a 1st–3rd postoperative day corrected VA ≥ 0.3.

B. Distribution of cataract surgeries in urban and suburban areas of Shanghai from 2006 to 2009 (Tables 2 and 3).

**Table 2:** Analysis of cataract surgeries between urban and suburban areas, 2006 to 2009

	**District**	**No. CS**	**Population**	**CSR**	**Surgical methods (%)**			**VA outcomes**	
			**million**		**Phaco**	**ECCE**	**others**	**A%**	**B%**
2006	Urban areas	24785	6.50	3813	90.65	8.04	1.31	98.74	86.91
	Suburban areas	6819	11.65	585	83.10	16.73	0.17	98.00	89.44
2007	Urban areas	27478	6.49	4234	91.08	7.67	1.25	98.41	85.68
	Suburban areas	7085	12.09	586	90.30	9.60	0.10	97.28	87.41
2008	Urban areas	32131	6.53	4921	94.69	4.34	0.97	97.47	87.91
	Suburban areas	6617	12.35	536	89.33	10.50	0.17	96.96	88.76
2009	Urban areas	35707	6.53	5468	95.56	3.30	1.14	98.15	87.34
	Suburban areas	6752	12.68	532	91.63	8.16	0.21	96.59	88.19

*Abbreviations* used: *No. CS* the number of cataract surgeries, *Phaco* phacoemulsification surgeries, *ECCE* extracapsular cataract extraction surgeries, *A%* percentage of patients with 1st–3rd postoperative day corrected Snellen visual acuity (VA) ≥ 0.05; *B%*, percentage of patients with 1st–3rd postoperative day corrected Snellen VA ≥ 0.3.

**Table 3:** Distribution of medical resources in urban and suburban areas of Shanghai, 2006 to 2009

**Year**	**District**	**No. hospitals**	**Distribution of No. CS**						**No. surgeons**	**No.CS/ year/surgeon**
			**>5000**	**2000–5000**	**1000–2000**	**500–1000**	**100–500**	**<100**		
2006	Urban areas	60	1	0	6	5	35	13	222	112
	Suburban areas	36	0	0	0	3	15	18	70	97
2007	Urban areas	61	1	1	6	5	31	17	234	117
	Suburban areas	36	0	0	0	3	16	17	75	94
2008	Urban areas	61	1	1	6	9	30	14	253	127
	Suburban areas	36	0	0	0	3	20	13	73	90
2009	Urban areas	61	1	5	3	8	31	13	263	136
	Suburban areas	36	0	0	0	4	19	13	75	90

Notes: In 2007, the southern branch of Shanghai First People’s Hospital, a medical center, was opened in Songjiang District; *No. CS*: the number of cataract surgeries.

In the most recent four years, the number of cataract surgeries in the city proper increased significantly, with the CSR reaching 5468 by 2009, which was 10-fold greater than that in the suburbs. Meanwhile, the number of cataract surgeries in the suburbs showed almost no changes during these years, with an actual decline from 21.58% in 2006 to 15.90% in 2009. However, the percentage of phacoemulsification surgeries performed in the suburbs increased from 83.10% to 91.63%, indicating that the gap in surgical methods between the two areas was steadily narrowing. In the Shanghai area, 70% of the population but only 20% of surgeons and 30% of cataract-surgery units, mostly in district hospitals, were in the suburbs. Furthermore, only 4 units performed more than 500 cataract surgeries, while 13 units performed less than 100 surgeries per year. Lastly, there were no significant differences in VA outcomes after surgery between the two areas.

C. Cataract surgery medical resource distribution in different types of medical institutes in Shanghai, 2006 to 2009 (Tables 4 and 5).

**Table 4:** Analysis of cataract surgeries in different medical institutions in Shanghai, 2006 to 2009

**Year**	**Type of cataract surgery unit**	**No. CS/year**	**Surgical methods (%)**			**VA outcomes**	
			**Phaco**	**ECCE**	**Others**	**A%**	**B%**
2006	Medical centers	17194	90.19	8.00	1.81	98.80	86.46
	District hospitals	12856	90.49	9.37	0.14	98.26	88.50
	Private hospitals	1554	63.90	35.59	0.51	98.91	89.83
2007	Medical centers	18930	91.45	6.87	1.68	98.65	86.07
	District hospitals	12794	94.06	5.79	0.15	97.30	86.38
	Private hospitals	2839	73.19	26.28	0.53	98.98	84.18
2008	Medical centers	21137	91.97	6.74	1.29	98.10	87.28
	District hospitals	12770	94.75	4.94	0.31	95.60	88.20
	Private hospitals	4841	99.11	0.74	0.15	99.07	91.03
2009	Medical centers	22218	93.13	5.17	1.70	97.82	86.94
	District hospitals	13610	95.56	4.14	0.30	97.27	87.41
	Private hospitals	6631	99.71	0.24	0.05	99.47	89.44

*Abbreviations* used: *No. CS* the number of cataract surgeries, *Phaco* phacoemulsification surgeries, *ECCE* extracapsular cataract extraction surgeries, *A%* percentage of patients with 1st–3rd postoperative day corrected Snellen visual acuity (VA) ≥ 0.05, *B%* percentage of patients with 1st–3rd postoperative day corrected Snellen VA ≥ 0.3.

**Table 5:** Cataract surgery medical resource distribution in different medical institutions in Shanghai, 2006 to 2009

**Year**	**Type of cataract surgery unit**	**No. surgeons**	**No. CS/ year/surgeon**	**No. surgery units**	**Distribution of No. CS**					
					**>5000**	**2000–5000**	**1000–2000**	**500–1000**	**100–500**	**<100**
2006	Medical centers	138	125	22	1	0	6	2	9	4
	District hospitals	137	94	68	0	0	0	3	48	17
	Private hospitals	17	91	6	0	0	0	1	2	3
2007	Medical centers	143	132	23	1	1	5	3	9	4
	District hospitals	143	89	68	0	0	0	4	35	29
	Private hospitals	23	123	6	0	0	1	1	3	1
2008	Medical centers	151	140	23	1	1	4	6	8	3
	District hospitals	145	88	68	0	0	0	5	38	25
	Private hospitals	30	161	6	0	0	2	1	2	1
2009	Medical centers	153	145	23	1	3	3	4	9	3
	District hospitals	153	89	68	0	0	0	6	38	24
	Private hospitals	32	207	6	0	2	0	2	1	1

*Abbreviation* used: *No. CS* the number of cataract surgeries.

Among almost 100 hospitals that were able to accept cataract surgeries, only 10% of them, mostly medical centers, performed an actual number of surgeries exceeding 1000. In the past four years, the actual number of surgeries in medical centers had increased by 29.22%. Meanwhile, private hospitals were evolving much more rapidly, with a greater than 300% increase in the number of surgeries per year. In 2009, the number of cataract surgeries performed within the six private hospitals accounted for 15.62% of the total, and an average of 207 cataract surgeries were performed by each surgeon, which exceeded the levels in medical centers by far. At the same time, the percentage of phacoemulsification surgeries increased considerably from 63.90% to 99.71%.

However, among 68 district hospitals, only 6 units performed more than 500 surgeries, with 30% of them performing less than 100. The number of cataract surgeries was nearly constant in the past four years; each surgeon was required to perform only 89 surgeries on average by 2009, even less than the data reported in 2006.

## Discussion

In 1999, the WHO and the International Association for Prevention of Blindness (IAPB) launched “Vision 2020: The Right to Sight” to eliminate avoidable blindness by the year 2020, which was actively supported by the Chinese government. Now, 12 years later, cataracts are still the leading cause of blindness in China. The CSR throughout China was only 670 in 2006, and 800 in 2008 [5]. Shanghai, as an economically and culturally developed city in China, has made some progress in blindness prevention in recent years. However, its CSR was only 2210 by the year 2009, which lags behind the level of 3000, the minimum necessary to achieve the WHO goals of “Vision 2020” [6]. A low number of cataract surgeries in suburban areas is the key factor that has significantly reduced the total CSR in Shanghai. Therefore, raising the CSR in suburban areas of Shanghai is crucial to increasing the total CSR in Shanghai.

According to literature reports, important barriers to cataract surgery in developing countries include the cost of surgery and IOL, lack of awareness, poor service, and long distances from surgical centers [7].

By 2009, the per capita disposable incomes of urban and rural residents in Shanghai were about US$4530 and US$1950 respectively. The cost of phacoemulsification with IOL implantation in Shanghai is about US$900, of which about US$600 is paid from Medicare. If the patient received a second eye cataract surgery, about US$800 was paid from Medicare. Therefore, suburban residents in Shanghai were able to afford the cost of cataract surgery.

One obvious indicator of poor quality of service is poor postoperative vision [7]. The data in our study showed no differences in outcome between the two areas. Thus, cost and poor quality service may not be the main reasons for a low CSR in suburban areas.

The current situation discloses an improper distribution of cataract surgery resources in Shanghai, assigning much fewer resources to suburban areas. To balance this improper distribution, the “5 + 3 + 1” project was initiated by the government in 2009 [8]. During the next few years, five tertiary hospitals will be established in Pudong, Minhang, Baoshan, Jiading, and Nanhui; three secondary level hospitals will be upgraded to tertiary B level hospitals in Chongming, Nanhui, and Qingpu; and one tertiary hospital will be relocated in Jinshan. According to the proposed schedule, it will require only 1 h for residents living in the suburbs to reach medical centers [9]. However, we are doubtful whether this project alone can raise the CSR in suburban Shanghai.

Although many more people live in the suburbs, the actual number of cataract surgeries per surgeon there was only 90, less than the average of 136 in the city proper by 2009. These data may indicate that many patients in suburban areas of Shanghai were not willing to have cataract surgery, so the surgical workload of surgeons there remained unsaturated. Due to culture restraints and inaccessibility to current information, we suspect that lack of awareness might be one of the most important barriers to cataract surgery in suburban areas of Shanghai. Some reports describing barriers to cataract surgery in suburban China substantiate this viewpoint [10-13]. Therefore, we propose that it should be a priority to raise awareness in the suburban population to better understand and be more willing to accept cataract surgery at the present time. Without this prerequisite, despite construction of more surgery units in the suburbs to reduce travel distances to surgical centers, a significant increase in the CSR may not be likely.

Last but not the least, the considerable growing rate of cataract surgeries in private hospitals in recent years should not be overlooked. Under similar conditions of outcome and cost, why more cataract patients prefer to have surgery performed in private cataract surgery units is an important question, and problem, that eye doctors in public hospitals should ponder.

Limitations are unavoidable in this study. Because collecting all long-term follow-up data for cataract surgery patients in Shanghai is impossible, the outcome of cataract surgery was only measured by the 1st–3rd postoperative day corrected VA, which may not provide insight into the long-term effects of cataract surgery in Shanghai. In addition, more detailed data regarding surgical complications should be collected in future studies. Another limitation is that the age distribution pattern in the population was not considered in this analysis because only the data of the registered population who is over 60 years old could be found in the Shanghai Statistical Yearbook.

## Conclusions

Although the level of economic development in Shanghai is in a world-leading position, its CSR remains less than that of social development and has not achieved the target suggested by the WHO. The low level of CSR in the suburbs and the small number of cataract surgeries in district hospitals are main factors that limit increasing the CSR in this city. Raising awareness in the suburban population is the first measure that we should take at the present time.

## Abbreviations

CSR: Cataract surgery rate; WHO: World health organization; GDPPC: Gross domestic product per capita; IOL: The intraocular lens; Phaco: Phacoemulsification; ECCE: Extracapsular cataract extraction; IPAB: International association for prevention of blindness.

## Competing interests

The authors declare that they have no competing interests.

## Authors’ contributions

MZHU designed the study, analyzed the data and wrote the article. JZHU conceived of the study, participated in its design and helped to draft the manuscript. LL and XH controlled the quality of all the data and participated in the design of the study. RZ performed the statistical analysis. HZ designed the study and proofed article. All authors read and approved the final manuscript.

## Pre-publication history

The pre-publication history for this paper can be accessed here:

http://www.biomedcentral.com/1471-2415/14/3/prepub
